# Pre-Transplant Hyperparathyroidism and Graft or Patient Outcomes After Kidney Transplantation

**DOI:** 10.3389/ti.2024.11916

**Published:** 2024-02-07

**Authors:** Fernanda Guedes Rodrigues, Willemijn Y. Van Der Plas, Camilo German Sotomayor, Amarens Van Der Vaart, Daan Kremer, Robert A. Pol, Schelto Kruijff, Ita Pfeferman Heilberg, Stephan J. L. Bakker, TransplantLines Investigators, Martin H. De Borst

**Affiliations:** ^1^ Division of Nephrology, Department of Internal Medicine, University Medical Center Groningen, University of Groningen, Groningen, Netherlands; ^2^ Nutrition Post Graduation Program, Universidade Federal de São Paulo (UNIFESP), São Paulo, Brazil; ^3^ Department of Surgery, Division of Surgical Oncology, University Medical Center Groningen, University of Groningen, Groningen, Netherlands; ^4^ Department of Surgery, Amsterdam UMC, University of Amsterdam, Amsterdam, Netherlands; ^5^ Department of Endocrinology, University of Groningen, University Medical Center Groningen, Groningen, Netherlands; ^6^ Department of Surgery, Martini Hospital Groningen, Groningen, Netherlands; ^7^ Department of Molecular Medicine and Surgery, Karolinska Institutet, Stockholm, Sweden; ^8^ Nephrology Division, Universidade Federal de São Paulo (UNIFESP), São Paulo, Brazil

**Keywords:** kidney transplantation, graft survival, delayed graft function, hyperparathyroidism, mineral metabolism

## Abstract

The impact of pre-transplant parathyroid hormone (PTH) levels on early or long-term kidney function after kidney transplantation is subject of debate. We assessed whether severe hyperparathyroidism is associated with delayed graft function (DGF), death-censored graft failure (DCGF), or all-cause mortality. In this single-center cohort study, we studied the relationship between PTH and other parameters related to bone and mineral metabolism, including serum alkaline phosphatase (ALP) at time of transplantation with the subsequent risk of DGF, DCGF and all-cause mortality using multivariable logistic and Cox regression analyses. In 1,576 kidney transplant recipients (51.6 ± 14.0 years, 57.3% male), severe hyperparathyroidism characterized by pre-transplant PTH ≥771 pg/mL (>9 times the upper limit) was present in 121 patients. During 5.2 [0.2–30.0] years follow-up, 278 (15.7%) patients developed DGF, 150 (9.9%) DCGF and 432 (28.6%) died. A higher pre-transplant PTH was not associated with DGF (HR 1.06 [0.90–1.25]), DCGF (HR 0.98 [0.87–1.13]), or all-cause mortality (HR 1.02 [0.93–1.11]). Results were consistent in sensitivity analyses. The same applied to other parameters related to bone and mineral metabolism, including ALP. Severe pre-transplant hyperparathyroidism was not associated with an increased risk of DGF, DCGF or all-cause mortality, not supporting the need of correction before kidney transplantation to improve graft or patient survival.

## Graphical Abstract

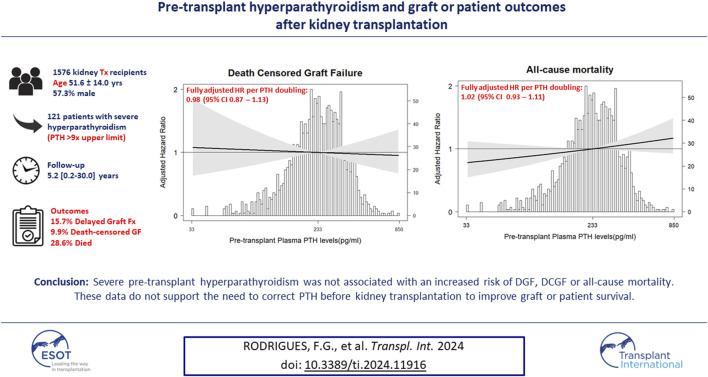

## Introduction

Hyperparathyroidism is a frequent complication of advanced chronic kidney disease (CKD) [1]. While a moderate increase in plasma parathyroid hormone (PTH) may be indicative of an appropriate compensatory response to maintain normal calcium balance, very high PTH levels have been associated with reduced quality of life and an increased risk of cardiovascular and bone disease and premature mortality in patients with kidney failure [2–4].

Kidney transplantation may at least in part resolve metabolic disturbances including hyperparathyroidism [5]. However, the presence of severe hyperparathyroidism at the time of transplantation may induce both short- and long-term adverse effects to the kidney. Early after successful kidney transplantation, persistently elevated levels of the phosphaturic hormones PTH and fibroblast growth factor-23 in the context of restored kidney function can induce high urinary concentrations of calcium and phosphate, which may lead to the deposition of calcium-phosphate and, consequently, acute tubular necrosis [6–8]. On the longer term, persistent or recurrent abnormalities in mineral metabolism have been associated with death-censored graft failure (DCGF), progression of vascular calcification and premature mortality [9, 10]. Similar to PTH, pretransplant serum total alkaline phosphatase, calcium and phosphorus levels have been associated with an increased risk of unfavorable outcomes after kidney transplantation [7, 9, 11, 12]. For these reasons, current Kidney Disease Improving Global Outcomes (KDIGO) guidelines suggest to not transplant patients with severe hyperparathyroidism until they are adequately treated, with PTH levels in the range of approximately 2–9 times the upper normal limit for the assay [13–15]. In contrast, the European Renal Association recommended in 2013 that a deceased donor allograft should not be refused only because of uncontrolled hyperparathyroidism in the recipient [15]. These conflicting recommendations urge for large cohort studies to examine the association between pre-transplant PTH level and clinically important post-transplant outcomes [13] including graft and patient outcomes.

Therefore, the aim of the present study was to assess whether patients with higher pre-transplant plasma PTH levels, and particularly those with severe hyperparathyroidism, have a higher risk of delayed graft function (DGF), death-censored graft function (DCGF), or all-cause mortality. We addressed this aim in a large contemporary cohort of kidney transplant recipients, and also studied associations of other mineral parameters (total alkaline phosphatase, calcium, and phosphate), measured before transplantation, with post-transplant outcomes.

## Materials and Methods

For the current study, all patients who underwent kidney transplantation at the University Medical Center Groningen (UMCG), Netherlands, between April/1986-December/2019 were considered eligible for inclusion. Of patients who had undergone multiple kidney transplantations, only data regarding the first kidney transplantation were used (*N* = 1,717). Patients with missing pre-transplant plasma PTH (*N* = 29) or if pre-transplant plasma PTH measurement was measured longer than 90 days before transplantation (*N* = 112) were excluded, leaving 1,576 patients for the DGF analysis. Furthermore, we excluded patients who developed graft failure or died within 3 months after transplantation (*N* = 67) [10], leaving 1,509 patients for the DCGF and all-cause mortality analysis (Supplementary Figure S1). The study protocol has been approved by the Institutional Review Board (METc 2014/077), was performed under the Strengthening the Reporting of Observational Studies in Epidemiology guidelines [16], adheres to the local UMCG Biobank Regulations, and is in accordance with the WMA Declarations of Helsinki and Istanbul.

### Laboratory Data

Routine laboratory measurements were extracted from the laboratory information system of the UMCG. Plasma PTH, calcium, phosphate, total alkaline phosphatase (ALP), creatinine, and albumin concentrations were measured at outpatient visits. Plasma calcium was corrected for albumin according to the following formula: corrected calcium (mg/dL) = measured calcium (md/dL)+0.025*(40–[albumin (g/dL)]). All routine measurements before March 2006 were performed on the Merck Mega Analyzer (Merck); measurements after March 2006 were performed on the Roche Modular (Roche Ltd.). Laboratory measurements prior to March 2006 were converted according to the equations [17] listed in Supplementary Table S1. The last PTH measurement prior to the kidney transplant procedure was used for analyses. Reference values for plasma-corrected calcium were 8.8–10.4 mg/dL (2.20–2.60 mmol/L) and for plasma phosphate 2.17–4.64 mg/dL (0.70–1.50 mmol/L) [10]. At each individual measurement, patients were classified as having hypo-, normo-, or hypercalcemia and hypo-, normo-, or hyperphosphatemia according to these definitions. Creatinine-based eGFR was calculated according to the CKD Epidemiology Collaboration Equation (EPI) equation [18, 19]. Primary cytomegalovirus (CMV) infection was defined as CMV viremia demonstrated by PCR in the absence of CMV-specific IgG antibodies. All other measurements were performed using standard laboratory techniques.

### Follow-Up

All patients who received a kidney transplant underwent a standardized follow-up regime. Patients received a standardized immunosuppression protocol, comprising triple therapy with tacrolimus or cyclosporine, in combination with mycophenolate mofetil and corticosteroids, as previously reported [20]. Shortly after transplantation, patients visit the outpatient department weekly. The frequency of visits is tapered to every 4–6 weeks during the first year after transplantation, and at least four times a year after the first year. End of follow-up was December 2020. Donor and recipient characteristics were collected as part of the TransplantLines registry [21]. The primary cause of kidney failure was categorized according to the European Renal Association Registry Coding System [22]. Acute rejection was defined according to the Banff criteria. There was no loss to follow-up.

### Study Endpoints

The three co-primary outcomes were DGF, defined as the need for dialysis within the first 7 days posttransplant, DCGF, defined as return to dialysis or re-transplantation, censored for death, and all-cause mortality. Up-to-date follow-up was warranted through the continuous surveillance system of the outpatient clinic.

### Statistical Analyses

Statistical analyses were performed using IBM SPSS version 23.0 (SPSS Inc., Chicago, IL). In all analyses *p* < 0.05 was considered significant. Variable distribution was evaluated by Kolmogorov Smirnov test. Categorical variables are presented as *n* (%), normally distributed variables as mean ± standard deviation (SD) and non-normally distributed variables as median with interquartile range (IQR). Skewed variables were log-transformed where appropriate. We handled remaining missing data for key variables using multiple imputation of variables with less than 10% missing data. Data of the following variables were imputed using multiple imputation by chained equations with five imputations: total alkaline phosphatase, calcium, phosphate, cold ischemia time, warm ischemia time, number of human leucocyte antigen (HLA) mismatches, body mass index (BMI), donor status, presence of diabetes, use of cinacalcet, and use of vitamin D. Results from analyses on each imputed data set were then pooled according to Rubin’s rules [23].

To analyze whether pre-transplant plasma PTH, ALP, calcium and phosphate were independently associated with DGF, DCGF and all-cause mortality, we performed logistic and Cox-proportional regression analyses, respectively. Plasma levels were analyzed as categorical variables and as (log-transformed) continuous variables. The proportional hazard assumption was tested using statistical tests and graphical diagnostics based on the scaled Schoenfeld residuals. For PTH, patients were clustered in three groups according to the KDIGO guidelines-recommended thresholds: ≤2 times (≤150 pg/mL), >2 and <9 times (>150 and <771 pg/mL), or ≥9 times (≥771 pg/mL) of the upper limit of normal for the assay [24], using the middle range as reference. For ALP, calcium and phosphate, patients were analyzed in quartiles. Since most patients had normal or elevated levels of ALP at time of transplantation, we defined the lowest quartile as reference category for this parameter. For calcium and phosphate, we used the second quartile as reference category, since both very high and very low levels may occur at transplantation and both might be associated with adverse outcomes. We performed multivariate Cox regression analyses, cumulatively adjusted for age and sex (Model 1), and further variables previously associated with outcomes after kidney transplantation and bone mineral metabolism such as primary cause of kidney failure, primary CMV infection, acute allograft rejection, dialysis vintage, preemptive transplant, number of HLA mismatches, donor age and sex, living donor status, cold and warm ischemia time, history of diabetes, body mass index, serum calcium, phosphate and albumin at transplantation time, cinacalcet, vitamin D use, history of parathyroidectomy and decade of transplantation (Model 2; Supplementary Figure S2). The associations of pre-transplant PTH levels with DGF, DCGF and mortality were further investigated using restricted cubic splines using fully adjusted models. We also evaluated whether pre-transplant plasma PTH and ALP contributed to prediction of mortality risk using ROC-curve analysis with determination of the area under the ROC-curve (AUC). Finally, we studied the relationship between PTH at 1 year post-transplantation and DCGF or mortality, both in continuous analysis and using quartiles (fully adjusted models as described above). In these analyses, patients who developed graft loss or died within the first year post-transplant were excluded.

Potential effect modification for the association between pre-transplant PTH and outcomes was explored using multiplicative interaction terms followed by prespecified subgroup analyses according to age, sex, use of vitamin D, use of cinacalcet, preemptive kidney transplant, dialysis vintage, donor status (living or deceased), serum calcium, phosphate and ALP. The *p*-values of interaction terms were considered significant when <0.05.

Finally, we performed sensitivity analyses for the DGF analysis, restricted to patients who received from a postmortal donor (as DGF is much more common after postmortal donor kidney transplantation), and for the mortality analyses after exclusion of individuals who died within the first year post-transplant, and restricted to patients transplanted after 2010.

## Results

### Baseline Characteristics

A total of 1,576 kidney transplant recipients (KTRs) (age 51.6 ± 14.0 years, 57.3% male) were included in the primary analyses. Baseline patient and transplant characteristics are presented in Table 1. In brief, donor age was 51.2 ± 13.5 years, 785 (49.8%) patients received a graft from a living donor, median (IQR) dialysis vintage was 18.0 (0–40.0) months, and 535 (33.9%) patients underwent a pre-emptive transplantation. Median (IQR) pre-transplant plasma PTH concentration was 231 (122–425) pg/mL, with 121 (7.7%) of patients presenting with plasma PTH ≥771 pg/mL (median 942 [838–1,236]pg/mL). Calcimimetics were used at time of transplant by 198 (12.6%) patients, and vitamin D analogs by 58% of patients. There were few missing data points (Supplementary Table S2).

**TABLE 1:** Baseline characteristics of the cohort.

Baseline characteristics	Total *n* = 1,576	PTH ≤150 pg/mL^a^ n = 496	PTH >150 < 771 pg/mL^b^ n = 959	PTH ≥771 pg/mL^c^ n = 121
Age at time of kidney transplantation, years	51.6 ± 14.0	51.7 ± 13.6	51.9 ± 14.2	47.8 ± 13.9
Sex (male), *n* (%)	903 (57.3)	271 (54.6)	556 (58.0)	76 (62.8)
Decade of transplantation				
1980–1989, *n* (%)	29 (1.8)	20 (4.0)	8 (0.8)	1 (0.8)
1990–1999, *n* (%)	87 (5.5)	53 (10.7)	30 (3.1)	4 (3.3)
2000–2009, *n* (%)	411 (26.1)	130 (26.2)	233 (24.9)	48 (39.7)
2010–2019, *n* (%)	1,049 (66.6)	293 (59.1)	688 (71.7)	68 (56.2)
BMI, kg/m^2^	26.2 ± 10.2	26.6 ± 4.7	27.1 ± 4.6	27.4 ± 5.0
*Primary kidney disease*				
Glomerulonephritis, *n* (%)	443 (28.1)	149 (30.0)	266 (27.7)	28 (23.1)
Interstitial nephritis, *n* (%)	264 (16.8)	67 (13.5)	173 (18.0)	14 (11.6)
Cystic kidney disease, *n* (%)	211 (13.4)	76 (15.3)	121 (12.6)	69 (57.0)
Diabetes Mellitus, *n* (%)	72 (4.6)	29 (5.8)	40 (4.2)	3 (2.5)
Renal vascular disease, excluding vasculitis, n (%)	82 (5.2)	32 (6.5)	48 (5.0)	2 (1.7)
Other congenital/hereditary kidney disease, *n* (%)	110 (7.0)	26 (5.2)	69 (7.2)	15 (12.4)
Other multisystem diseases, *n* (%)	78 (4.9)	24 (4.8)	50 (5.2)	4 (3.3)
Other, *n* (%)	59 (3.7)	15 (3.0)	33 (3.4)	11 (9.1)
Unknown, *n* (%)	257 (16.3)	78 (15.7)	159 (16.6)	20 (16.5)
*Medication use*				
Cinacalcet, *n* (%)	198 (12.6)	32 (6.4)	123 (12.8)	43 (35.5)
Vitamin D, *n* (%)	909 (57.7)	238 (48.0)	595 (62.0)	76 (62.8)
Antihypertensives, *n* (%)	1,270 (80.6)	373 (75.2)	798 (83.2)	99 (81.8)
Statins, *n* (%)	427 (27.1)	117 (23.6)	281 (29.3)	29 (24.0)
Laboratory parameters				
PTH, pg/mL	231 (122–425)	85 (49–117)	311 (215–451)	942 (838–1,236)
Calcium, mg/dL	9.6 ± 0.8	9.7 ± 0.8	9.5 ± 0.7	9.6 ± 0.8
Phosphate, mg/dL	4.8 ± 1.5	4.6 ± 1.4	4.9 ± 1.5	5.3 ± 1.4
Total alkaline phosphatase, U/L	100 (80–140)	69 (55–89)	79 (62–102)	111 (83–158)
Albumin, g/dL	4.3 ± 0.5	4.3 ± 0.5	4.3 ± 0.4	4.3 ± 0.4
Transplantation data				
Pre-emptive transplant, *n* (%)	535 (33.9)	154 (31.0)	345 (36.0)	36 (29.8)
Dialysis vintage, months	18.0 (0–40.0)	18.0 (0–38.0)	16.0 (0–39.0)	34.0 (0–63.0)
Living donor, *n* (%)	785 (49.8)	243 (49.0)	498 (51.9)	44 (36.4)
Donor age, years	51.2 ± 13.5	50.0 ± 14.4	52.5 ± 13.0	48.0 ± 13.3
Donor sex (male), *n* (%)	808 (51.2)	252 (50.8)	494 (51.5)	62 (51.2)
Number of HLA mismatches (A/B/DR)	2.0 (1.0–3.0)	3.0 (2.0–4.0)	3.0 (2.0–4.0)	3.0 (2.0–4.0)
Cold ischemia time, hours	5.0 (2.0–14.0)	7.7 (2.6–15.3)	3.7 (2.6–13.9)	11.8 (2.9–16.3)
Second warm ischemia time, minutes	41.4 ± 12.0	41.2 ± 11.9	41.5 ± 12.2	40.6 ± 10.5
Acute rejection, *n* (%)	256 (16.2)	77 (15.5)	163 (17.0)	16 (13.2)
CMV infection, *n* (%)	687 (43.5)	224 (45.2)	415 (43.3)	48 (39.7)

Categories correspond to <2x^a^, 2–9x^b^, and >9x^c^ upper limit of normal for the assay.

Data are presented as mean ± standard deviation (SD), median (IQR) or number (%). Abbreviations: BMI, body mass index; PTH, parathyroid hormone; HLA, human leukocyte antigen; CMV, cytomegalovirus.

### Pre-Transplant Plasma PTH Levels and Post-transplant Outcomes

**FIGURE 1:**
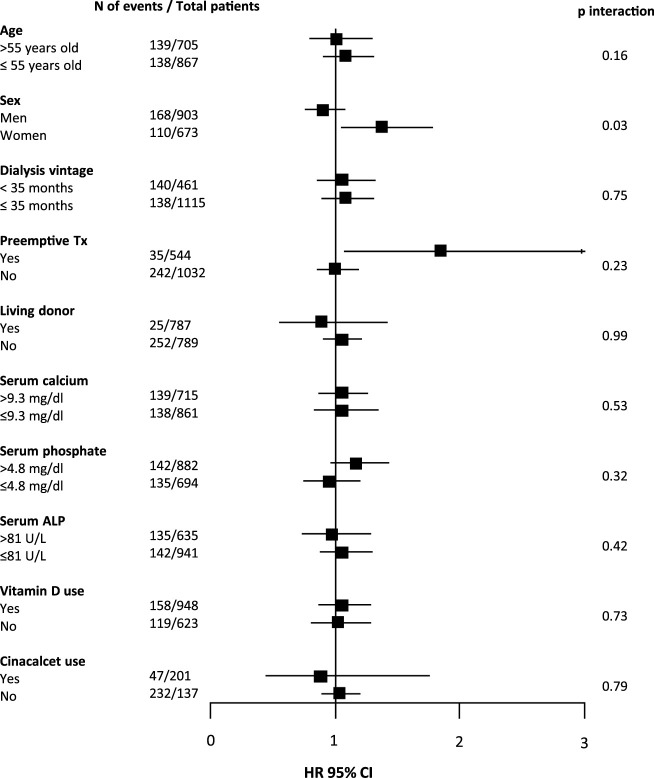
Forest plot showing associations between pre-transplant plasma PTH level (per doubling) and delayed graft function (DGF) according to parameters at time of transplantation. In total, 278 (16.7%) kidney transplant recipients developed DGF. Abbreviations: N, number; Tx, transplant; ALP, alkaline phosphatase; HR, hazard ratio.

As shown in Table 2, 278 (17.6%) patients developed DGF. Upon fully adjusted logistic regression analysis with pre-transplant PTH as a continuous variable, no significant association was found with DGF. When analyzing patients in three groups (≤2 times, >2 and <9 times, ≥9 times the upper limit of normal for the assay of plasma PTH, corresponding with ≤150 pg/mL, >150 and <771 pg/mL, and ≥771 pg/mL, respectively), patients with pre-transplant PTH >771 pg/mL had a risk of DGF that was comparable to the reference group (Table 2). Interaction analysis revealed significant effect modification by sex (P-interaction = 0.03), as shown in Figure 1. The incidence of DGF was similar among men (18.6%) and women (16.3%, *p* = 0.15 vs. men). The fully adjusted association between plasma PTH and DGF was significant among women (HR 1.37 [95% CI 1.05–1.78], *p* = 0.02), but not among men. In a sensitivity analysis restricted to women who received a graft from a postmortal donor, the association between low PTH levels and DGF did not persist (HR 1.14 [95% CI 0.89–1.46], *p* = 0.30).

**TABLE 2:** Association of pre-transplant PTH plasma levels with risk of DGF, DCGF and all-cause mortality.

Pre-transplant plasma PTH (pg/mL)	Events	Model 1: Adjusted for age + sex		Model 2: Fully adjusted	
		HR (95% CI)	*p*	HR (95% CI)	*p*
DGF					
Pre-KTx PTH, per doubling	**278/1,576**	1.23 (1.07–1.42)	<0.01	1.06 (0.90–1.25)	0.48
Pre-KTx PTH (pg/mL), groups					
PTH ≤150 pg/mL^a^	69/496	1.40 (1.03–1.89)	0.09 (p-trend)	0.82 (0.50–1.35)	0.35 (p-trend)
PTH >150 < 771 pg/mL	189/959	Reference		Reference	
PTH ≥771 pg/mL^b^	20/121	1.19 (0.69–2.05)		0.56 (0.24–1.32)	
DCGF					
Pre-KTx PTH, per doubling	**150/1,509**	0.98 (0.89–1.08)	0.68	0.98 (0.87–1.13)	0.86
Pre-KTx PTH (pg/mL), groups					
PTH ≤150 pg/mL^a^	56/475	0.98 (0.70–1.39)	0.78 (p-trend)	1.03 (0.62–1.67)	0.98 (p-trend)
PTH >150 < 771 pg/mL	83/921	Reference		Reference	
PTH ≥771 pg/mL^b^	15/113	0.80 (0.43–1.50)		1.01 (0.45–2.25)	
All-cause mortality					
Pre-KTx PTH, per doubling	**432/1,509**	1.06 (0.99–1.12)	0.17	1.02 (0.93–1.11)	0.66
Pre-KTx PTH (pg/mL), groups					
PTH ≤150 pg/mL^a^	157/485	0.99 (0.66–1.51)	0.14 (p-trend)	0.84 (0.63–1.12)	0.10 (p-trend)
PTH >150 < 771 pg/mL	248/921	Reference		Reference	
PTH ≥771 pg/mL^b^	27/113	1.22 (0.82–1.81)		0.56 (0.31–1.03)	

^a^ 2X of upper limit of normal for assay.

^b^ 9X of upper limit of normal for assay.

Model 1–adjusted for age, sex; Model 2–Model 1 + Primary kidney disease, cytomegalovirus (CMV) infection, acute allograft rejection, dialysis vintage, preemptive transplant, number of HLA, mismatches, donor age and sex, living donor status, cold and warm ischemia time, history of diabetes, body mass index, serum calcium, phosphate, total alkaline phosphatase and albumin at time of transplantation, cinacalcet and vitamin D use, history of parathyroidectomy and decade of transplantation. Abbreviations: DGF, delayed graft function; DCGF, death censored graft failure; PTH, parathyroid hormone; KTx, kidney transplantation; HR, hazard ratio; CI, confidence interval.

**FIGURE 2:**
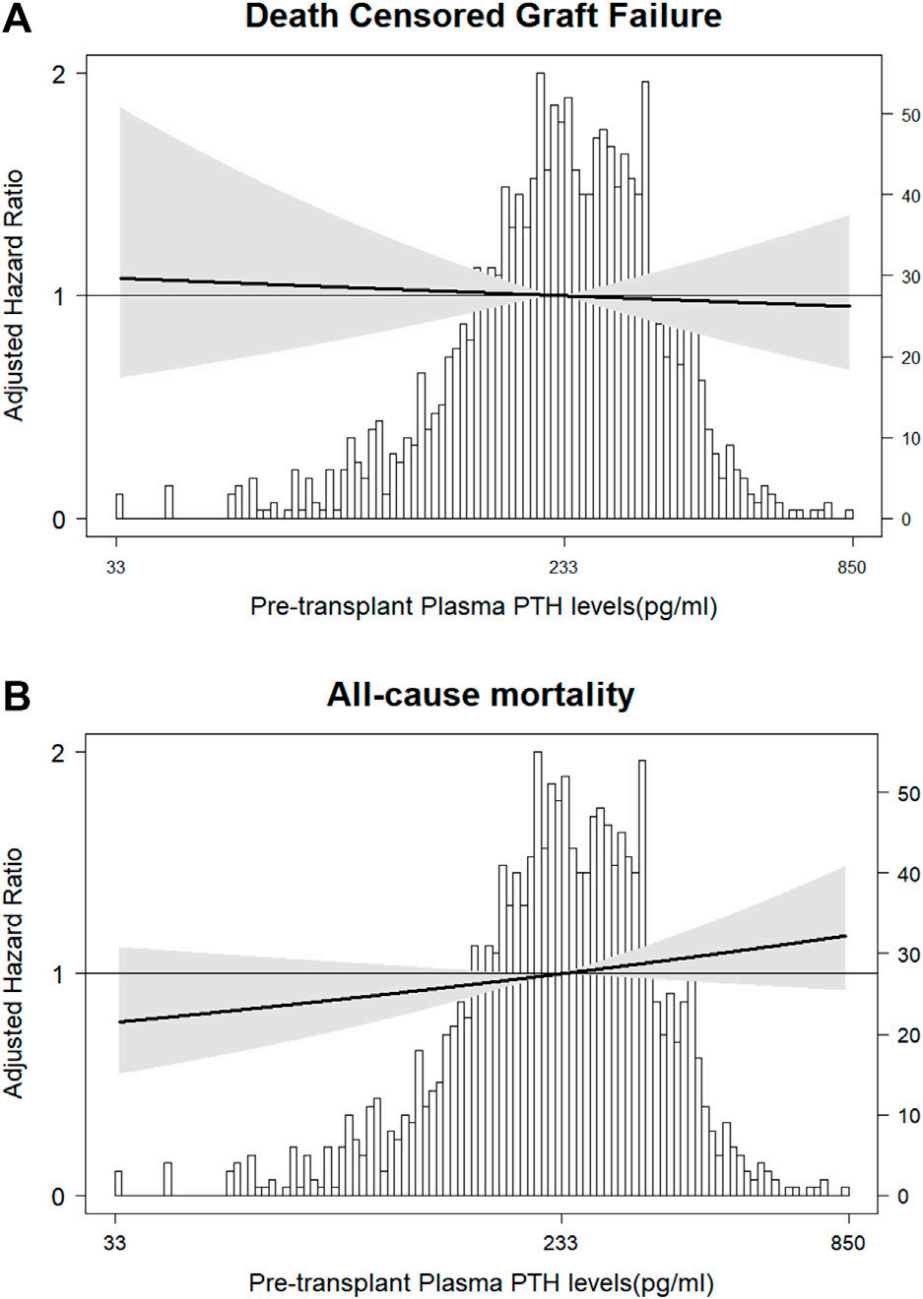
Association of pre-transplant plasma PTH with risk of **(A)** DCGF and **(B)** all-cause mortality. The solid lines represent the fully adjusted hazard ratios (HRs) for DCGF (Cox regression Model 2) and all-cause mortality (Cox regression Model 2). The grey areas represent the 95% confidence intervals of the HRs.

**FIGURE 3:**
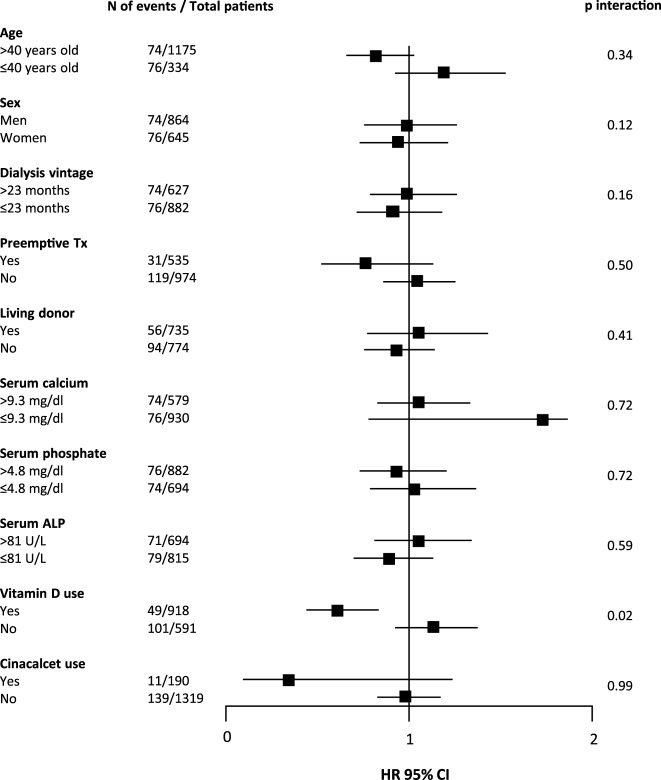
Forest plot showing associations between pre-transplant plasma PTH levels (per doubling) and death censored graft function (DCGF) according to parameters at time of transplantation. In total, 150 (9.9%) kidney transplant recipients developed DCGF. Abbreviations: N, number; Tx, transplant; ALP, alkaline phosphatase; HR, hazard ratio.

During median follow-up of 5.0 (range 0.2–29.5) years, 150 (9.9%) patients developed DCGF. In fully adjusted Cox regression analyses, pre-transplant plasma PTH levels were not associated with DCGF (Figure 2A). As shown in Table 2, patients with pre-transplant PTH ≤150 pg/mL or PTH >771 pg/mL had a risk that was comparable to the reference group (HR 0.98 [95% CI 0.87–1.67], *p* = 0.85; HR 1.01 [95% CI 0.45–2.25], *p* = 0.59, respectively). No significant effect modification was observed (Figure 3). Sensitivity analyses after exclusion of 39 individuals who developed DCGF within the first year after transplantation (*N* = 111, fully adjusted HR 0.94 [95% CI 0.83–1.08], *p* = 0.45) yielded similar results. At 1 year post-transplantation, the median PTH level was 110.5 (72.4–168.3) pg/mL. A higher PTH level at 1 year after transplantation was associated with an increased risk of DCGF (fully adjusted HR 1.31 [95% CI 1.03–1.69], *p* = 0.03) in continuous analysis (Supplementary Table S3). Patients in the highest quartile of PTH levels at 1 year post-transplant also had an increased risk of DCGF when compared with the lowest quartile (HR 2.64 [95% CI 1.21–5.80], *p* = 0.02).

During median follow-up of 5.2 (range 0.2–29.5) years, 432 (28.6%) patients died. In fully adjusted Cox regression analyses, pre-transplant plasma PTH levels were not associated with all-cause mortality (Table 2; Figure 2B). There was no significant effect modification in interaction analyses (Supplementary Figure S3). Sensitivity analyses after exclusion of 30 individuals who died within the first year post-transplant (*N* = 402, fully adjusted HR 1.03 [95% CI 0.95–1.12], *p* = 0.50), restricted to patients who were transplanted after 2010 (*N* = 197, HR 1.11 [95% CI 0.97–1.27], *p* = 0.12), or after exclusion of preemptive transplantation (*N* = 94, HR 0.99 [95% CI 0.82–1.19], *p* = 0.91) yielded similar results. An additional analysis in a limited number of patients with available PTH data from 6 to 12 months prior to transplantation (*N* = 187) showed no significant association with all-cause mortality (HR 0.78 [95% CI 0.55–1.16], *p* = 0.23). Finally, PTH at 1 year after transplantation showed a trend towards association with all-cause mortality (fully adjusted HR 1.14 [95% CI 0.99–1.32], *p* = 0.05) upon continuous analysis; patients in the highest PTH quartile (at 1 year post-transplant) had a higher risk of all-cause mortality than those in the lowest quartile (HR 2.64 [95% CI 2.20–5.81], *p* = 0.02).

### Pre-Transplant ALP, Calcium, and Phosphate and Post-transplant Outcomes

Subsequently, we analyzed the associations of pre-transplant serum ALP levels with risk of DGF, DCGF and all-cause mortality. There were no significant associations between serum ALP and risk of DGF, or DCGF (Supplementary Table S4). In age- and sex-adjusted analyses, higher serum ALP was associated with an increased risk of all-cause mortality, but this association lost significance upon multivariable adjustment. Compared with patients in the first quartile of pre-transplant serum ALP (52.0 [46.0–57.0]U/L), patients in fourth quartile with median pre-transplant serum ALP of 126.0 [110.8–158.0]U/L had a higher risk of all-cause mortality in fully adjusted model (HR 1.27 [95% CI 1.00–1.87]). There was no effect modification by pre-transplant dialysis status (preemptive or not) in interaction analysis (HR 1.30 [95% CI 0.72–2.32], *p* = 0.39). ALP, either alone or combined with PTH, did not change risk prediction for mortality when added to a model with established risk factors (Table 3). Plasma calcium was not significantly associated with the risk of DGF, DCGF, or all-cause mortality (Supplementary Table S5). Compared with patients in the second quartile of pre-transplant serum phosphate (4.2 [3.8–4.7]mg/dL), those in the fourth quartile with median pre-transplant serum phosphate of 6.5 [5.7–12.9]mg/dL had an increased risk of DGF in the fully adjusted model 2.21 [95% CI 1.21–4.03, *p* < 0.01] (Supplementary Table S6). Interaction analysis revealed significant effect modification by donor status (P-interaction<0.001). The fully adjusted association between plasma phosphate and DGF was significant among recipients of post-mortal draft (HR 1.5 [95% CI 1.03–2.33], *p* = 0.03), but not among recipients from a living donor. Plasma phosphate at transplantation was not associated with DCGF or mortality.

**TABLE 3:** Receiver operating characteristic (ROC) curve analysis for all-cause mortality.

	AUC	SE	95% CI	*p*-value (for change)^a^
Model 1	0.752	0.015	0.723–0.780	—
Model 2	0.752	0.015	0.723–0.780	0.61
Model 3	0.763	0.015	0.732–0.793	0.31
Model 4	0.763	0.015	0.733–0.793	0.45

AUC, area under curve; SE, standardized error; CI, confidence interval.

^a^ Versus Model 1.

Model 1: recipient age, corrected serum calcium, serum phosphate, serum albumin, preemptive transplantation, donor age, total cold ischemia, vitamin D use. Model 2: Model 1 + plasma PTH, at transplantation time. Model 3: Model 1 + serum ALP, at transplantation time. Model 4: Model 1 + plasma PTH, and ALP, at transplantation time.

### Post-Transplant Course of Plasma PTH, Calcium and Phosphate

**FIGURE 4:**
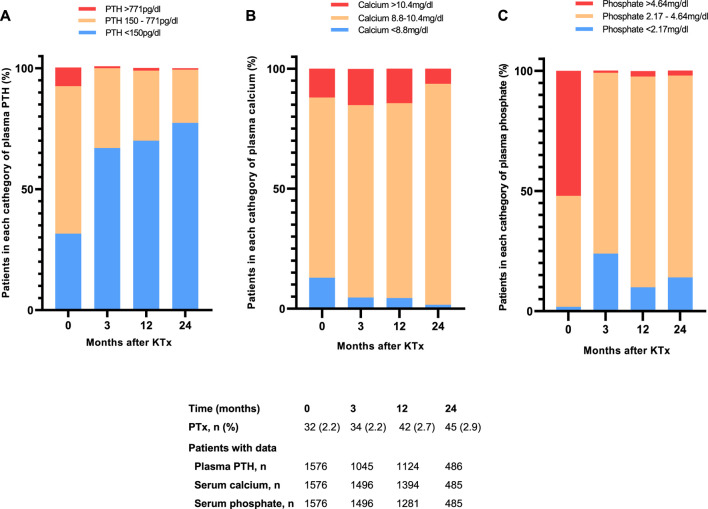
Distribution of proportion of patients with median plasma **(A)** PTH **(B)** calcium and **(C)** phosphate within, below or above the reference range for the first 24 months after kidney transplantation. Values are expressed as percentages. Abbreviations: PTH, parathyroid hormone; PTx, parathyroidectomy.

From 121 patients with severe hyperparathyroidism before transplantation, only 12 (10%) remained with elevated PTH (≥771 pg/mL) at 1 year post-transplant (Figure 4A). At baseline, almost one-third of patients had a calcium value outside the reference (13% hypocalcemia and 12% hypercalcemia). At 1 year after transplantation, 4.2% of patients presented with hypocalcemia, while 14.4% presented with hypercalcemia (Figure 4B). At time of transplantation more than half of patients had hyperphosphatemia (873 [55.6%] patients). At 1 year after transplantation, the prevalence of hyperphosphatemia had decreased to 3%. On the other hand, hypophosphatemia had a prevalence of 2% at baseline and of 10% at 1 year after transplantation (Figure 4C).

## Discussion

In this cohort of 1,576 primary stable kidney transplant recipients, we observed no associations between pre-transplant serum PTH levels and risk of DCGF, DGF and all-cause mortality in the primary analyses. Further, one-year post-transplant PTH levels were associated with DCGF and all-cause mortality. Interestingly, pre-transplant serum ALP levels higher than 126.0 (110.8–158.0) U/L were associated with higher risk of all-cause mortality, while serum phosphate levels higher than 6.5 (5.7–12.9) mg/dL were associated with higher risk of DGF; serum calcium levels at transplantation were not associated with post-transplant outcomes.

Previous studies regarding the potential risk of (severe) hyperparathyroidism at time of transplantation have shown conflicting results. In line with our findings, a previous large study found no associations with graft failure or mortality [9]. While this study only included patients that had been on chronic dialysis before transplantation, our study extends their findings by also including pre-emptive transplantations. A prior study from the same group also did not find a significant relationship between PTH and allograft loss and death [25]. In contrast, another study with a smaller sample size did find that a higher pre-transplant plasma PTH level was associated with a higher risk of death-censored graft failure (DCGF), but not with DGF or premature mortality [26]. Our finding that PTH at 1 year post-transplant was associated with DCGF and mortality is in line with prior studies [27–29], underscoring the importance of closely monitoring PTH levels after kidney transplantation so that patients with persistent or new-onset HPT post-transplant can be treated appropriately. Furthermore, it is important to consider other outcomes beyond graft and patient outcomes. While, to the best of our knowledge, no prior studies addressed the association between PTH at transplantation and fractures after kidney transplantation, on the other hand, post-transplant hyperparathyroidism has been associated with an increased risk of fractures [30], and correction of hyperparathyroidism by parathyroidectomy improved bone mineral density (BMD) [31]. Unfortunately, we could not address these associations in the current study as data on fractures or BMD were unavailable.

Interestingly, we found a significant association between higher PTH levels and DGF in women, but not in men. It has been described in experimental studies that female mice display greater tolerance for ischemia-reperfusion injury in multiple organs, including the kidney, and that estrogen may play a role in this protective mechanism [32–34]. In humans, a large cohort study suggested that this sex-dependent response to injury may have clinical implications for DGF after kidney transplantation [35]. In that study, the risk of DGF was significantly higher in male recipients, even after adjusting for potential confounders [35]. In the present study, we only observed a small and non-significant difference in incidence of DGF in men vs. women (18.3% vs. 16.1%, *p* = 0.15). At the same time, the apparent sex-specific association of PTH with DGF did not translate into a sex-specific (or overall) association with DCGF, and it lost significance in a sensitivity analysis restricted to patients with a postmortal donor. Therefore, our results on a potential sex-specific association between PTH and DGF require confirmation by an independent study, and the implications for clinical practice could well be limited.

Our study revealed an association between higher pre-transplant serum ALP and an increased risk of mortality after kidney transplantation. This observation is in line with two prior studies, one from the United States [9] and one from Korea [11]. In the study of Molnar et al. [9], it was suggested that the association between ALP and mortality could be driven by high-bone turnover during the dialysis period, which may influence mortality risk after transplantation. However, in our study, more than one-third of the patients received a transplant before requiring dialysis, and we found no effect modification of the association between ALP and mortality by pre-transplant dialysis status (HR 0.94 [95% CI 0.87–1.01], *p* = 0.08; data not shown in tables).

Whether pre-transplant plasma calcium is associated with adverse post-transplant outcomes is controversial. In the present study, we found no association between pre-transplant plasma calcium and DGF, DCGF or all-cause mortality. These results are in line with one previous study [36], while another study did show an independent association between serum calcium and DGF [7]. Interestingly, Molnar et al [9] found that high pre-transplant serum calcium levels (>9.5 mg/dL) were associated with a lower risk of graft loss, and hypothesized that this protective effect could be related to the vitamin D use. However, interaction analysis in our study did not reveal any effect modification by vitamin D use for the association between calcium and DCGF, which in itself also did not reach statistical significance (HR 0.98 [95% CI 0.33–2.90], *p* = 0.98).

Our findings show that a higher plasma phosphate level was associated with an increased risk of DGF. Although this result is in contrast with two prior studies that had a null outcome [37, 38], hyperphosphatemia could increase the risk of DGF through tubular deposition of calcium-phosphate crystals, leading to tubular obstruction and subsequent tubular injury, inflammation, and endothelial cell damage [39].

In our cohort, severe hyperparathyroidism at transplantation was present in only a small fraction of the population (7.7%). Furthermore, we found persistent hypercalcemia in 14.4% and hypophosphatemia in 10% of kidney transplant recipients at 12 months following transplantation. Although previous data reported the persistence of hyperparathyroidism ranging between 17% and 90% of transplanted patients, it is important to mention the use of different approaches to classify hyperparathyroidism [37–40]. Persistent hypercalcemia after kidney transplantation is relatively common with a prevalence ranging between 10% and 12% [10, 40, 41]. Although high levels of serum calcium could be the result of a persistent hyperparathyroidism, adynamic bone disease in combination with tubular reabsorption of calcium could be another cause of hypercalcemia after transplantation, and so may the use of calcium or vitamin D supplements [42]. The occurrence of hypophosphatemia following kidney transplantation is well described in the literature since during the initial post-transplant period, the accumulated plasma levels of PTH and FGF-23, together with the restored renal excretory capacity, stimulate phosphate excretion [43, 44]. Clearly, the generally improved abnormalities in mineral metabolism may be partly driven by the fact that affected patients received treatment with calcimimetics or underwent parathyroidectomy [45].

Our study has several limitations and strengths. The observational nature of this study leaves the possibility of residual confounding. PTH measurements after 2006 were converted using an in-house established conversion formula, which could be considered a limitation even though adjustment for transplant era did not influence the results and a sensitivity analysis did not suggest that the change in assays affected our findings. The repeated measures could have led to selection bias since patients with abnormal values may have been more frequently tested; on the other hand, these patients were also at higher risk to die within the first 2 years after transplantation. The lack of data on fractures, bone density measurements or bone biopsies, which would have allowed us to investigate specific bone outcomes are another limitation. The small number of patients with very high PTH levels (>9x the upper limit of normal assay) can be also considered as a limitation, although it likely does reflect practice in our center similar to many centers elsewhere in the world. The population was predominantly Caucasian, which calls for prudence when extrapolating these results to different populations. On the other hand, strengths include the large sample size, good characterization allowing for adequate adjustment and sensitivity analyses, external validity [46], complete follow-up which was longer than previous studies [9, 26], and clinically relevant endpoints.

In conclusion, in this large contemporary cohort of kidney transplant recipients, we found no association between severe hyperparathyroidism at the time of transplantation and the risk of DCGF or all-cause mortality. The observation that higher PTH levels are associated with an increased risk of DGF in women, but not in men, requires further investigation. Our finding that PTH levels at 1 year post-transplant were associated with DCGF and mortality underscores the importance of closely monitoring patients after transplantation to provide adequate treatment for persistent or new-onset hyperparathyroidism. Overall, although further studies are needed to address the impact on bone outcomes, our findings do not support the requirement of a pre-transplant parathyroidectomy to improve graft or patient survival in transplant candidates with severe hyperparathyroidism.

## Data Availability

The data supporting the findings of this study are available upon reasonable request.
