# Supplementary material for: Pre-Transplant Hyperparathyroidism and Graft or Patient Outcomes After Kidney Transplantation
Source: Transpl Int. 2024 Feb 7;37:11916. doi: 10.3389/ti.2024.11916 (PMC10880800; doi:10.3389/ti.2024.11916)
Supplement: Supplementary file 1 [file DataSheet1.PDF]

Supplementary material

Supplementary Tables

Supplementary Table 1. Conversion equations for results of laboratory measurements

Supplementary Table 2. Missing data

Supplementary Table 3. Associations of 1 year post-transplant PTH levels with risk of DCGF and all-cause mortality

Supplementary Table 4. Associations of pre-transplant total alkaline phosphatase levels with risk of DGF, DCGF and all-cause mortality

Supplementary Table 5. Association of pre-transplant (corrected) calcium levels with risk of DGF, DCGF and all-cause mortality

Supplementary Table 6. Association of pre-transplant phosphate levels with risk of DGF, DCGF and all-cause mortality

Supplementary Figures

Supplementary Figure 1. Diagram flowchart of patients inclusion

Supplementary Figure 2. Acyclic graph representing direct and indirect paths between variables included in multivariable models. A- Acyclic graph for Delayed Graft Function; B- Acyclic graph for Death Censored Graft Failure; C- Acyclic graph for all-cause mortality.

Supplementary Figure 3. Forest plot showing fully adjusted associations between pre-transplant plasma PTH levels and all-cause mortality according to subgroups and their interaction terms.

STROBE Statement - checklist of items that should be included in reports of observational studies

Supplementary Table 1. Conversion equations for results of laboratory measurements

**Measurement**	**Conversion equation**
Serum phosphate	$Y^a = (X^b - 0.03) / 0.97$
Serum creatinine	$Y^a = (X^b - 8) / 1.07$
Serum albumin	$Y^a = (X^b + 6) / 1.132$
Serum calcium	No differences between methods
Urinary protein	$Y^a = (X^b + 0.05 / 1.403)$
Serum PTH	$Y^c = (1.27 * X^d) + 0.5$

PTH, parathyroid hormone

[a] Roche Modular (Roche Ltd., Mannheim, Germany)

[b] Merck Mega Analyzer (Merck, Darmstadt, Germany)

[c] Immulite 2500 (Siemens Healthcare Diagnostics, Deerfield, IL, USA)

[d] ILMA (Advantage, Nichols Institute Diagnostics, CA, USA)

Conversion equations are presented in SI units.

Supplementary Table 2. Missing data

Variable	Missings, n (%)
Age at time of kidney transplantation	0 (0)
Decade of transplantation	0 (0)
BMI	181 (11.4)
Primary kidney disease	0 (0)
**Medication use**	
Cinacalcet	24 (1.5)
Vitamin D	100 (6.3)
Antihypertensives	77 (4.9)
Statins	96 (6.1)
**Laboratory parameters**	
PTH	0 (0)
Calcium	4 (0.3)
Phosphate	6 (0.4)
Alkaline phosphatase	335 (21.3)
Albumin	1 (0.1)
**Transplantation data**	
Pre-emptive transplant	0 (0)
Dialysis vintage	0 (0)
Donor status (living/deceased)	5 (0.3)
Donor age	7 (0.4)
Donor sex	4 (0.3)
Number of HLA mismatches (A/B/DR)	11 (0.7)
Cold ischemia time	71 (4.5)
Second warm ischemia time	8 (0.5)
Acute rejection, n(%)	0 (0)
CMV infection, n(%)	250 (15.9)

Abbreviations: BMI, body mass index; PTH, parathyroid hormone; HLA, human leukocyte antigen.

Supplementary Table 3. Associations of 1 year post-transplant PTH levels with risk of DCGF and all-cause mortality

**Pre-transplant Serum ALP level (U/L)**	**Events**	**Model 1**		**Model 2**	
		**HR (95% CI)**	***p***	**HR (95% CI)**	***p***
**DCGF**					
1 year post-KTx PTH, per doubling	**98/1109**	1.20 (1.01 – 1.44)	0.04	1.31 (1.03 – 1.69)	0.03
1 year post-KTx PTH (pg/ml), quartiles					
Q1, PTH 56 (43 – 65) pg/ml	17/302	Reference		Reference	
Q2, PTH 99 (88 – 104) pg/ml	17/391	1.21 (0.62 – 2.38)	0.07	0.85 (0.33 – 2.20)	0.02
Q3, PTH 146 (135 – 158) pg/ml	28/298	1.78 (0.97 – 3.26)	(p-trend)	1.31 (0.55 – 3.16)	(p-trend)
Q4, PTH 249 (196 – 332) pg/ml	36/289	2.00 (1.12 – 3.57)		2.64 (1.21 – 5.80)	
**All-cause mortality**					
1 year post-KTx PTH, per doubling	**287/1109**	1.14 (1.03 – 1.27)	<0.01	1.14 (0.99 – 1.32)	0.05
1 year post-KTx PTH (pg/ml), quartiles					
Q1, PTH 56 (43 – 65) pg/ml	53/302	Reference		Reference	
Q2, PTH 99 (88 – 104) pg/ml	57/291	1.21 (0.83 – 1.78)	0.04	0.85 (0.22 – 2.21)	0.02
Q3, PTH 146 (135 – 158) pg/ml	82/298	1.44 (1.01 – 2.04)	(p-trend)	1.31 (0.55 – 3.16)	(p-trend)
Q4, PTH 249 (196 – 332) pg/ml	95/289	1.60 (1.14 – 2.24)		2.64 (2.20 – 5.81)	

Model 1 – adjusted for age, sex; Model 2 – Model 1 + Primary cytomegalovirus (CMV) infection, acute allograft rejection, dialysis vintage, preemptive transplant, number of HLA mismatches, donor age and sex, living donor status, cold and warm ischemia time, history of diabetes, body mass index, serum calcium, phosphate, PTH and albumin at one year after transplantation, cinacalcet and vitamin D use, history of parathyroidectomy and decade of transplantation. Abbreviations: DGF, delayed graft function; DCGF, death censored graft failure; ALP, alkaline phosphatase; KTx, kidney transplantation; HR, hazard ratio; CI, confidence interval.

Supplementary Table 4. Associations of pre-transplant ALP levels with risk of DGF, DCGF and all-cause mortality

**Pre-transplant Serum ALP level (U/L)**	**Events**	**Model 1**		**Model 2**	
		**HR (95% CI)**	***p***	**HR (95% CI)**	***p***
**DGF**					
Pre-KTx ALP, per doubling	**241/1241**	1.11 (0.81 – 1.53)	0.52	1.15 (0.77 – 1.72)	0.50
Pre-KTx ALP (U/L), quartiles					
Q1, ALP 52 (46 – 57) U/L	53/317	Reference		Reference	
Q2, ALP 69 (65 – 72) U/L	47/299	0.80 (0.54 – 1.19)	0.74	0.79 (0.52 – 1.20)	0.70
Q3, ALP 87 (82 – 93) U/L	60/311	0.90 (0.62 – 1.31)	(p-trend)	0.82 (0.54 – 1.24)	(p-trend)
Q4, ALP 126 (110 – 158) U/L	81/314	0.88 (0.61 – 1.27)		0.85 (0.56 – 1.29)	
**DCGF**					
Pre-KTx ALP, per doubling	**143/1180**	1.25 (0.86 – 1.67)	0.25	0.91 (0.50 – 1.68)	0.77
Pre-KTx ALP (U/L), quartiles					
Q1, ALP 52 (46 – 57) U/L	34/302	Reference		Reference	
Q2, ALP 69 (65 – 72) U/L	38/391	1.26 (0.79 – 2.00)	0.73	0.94 (0.50 – 1.75)	0.35
Q3, ALP 87 (82 – 93) U/L	36/298	1.28 (0.80 – 2.04)	(p-trend)	1.45 (0.75 – 2.81)	(p-trend)
Q4, ALP 126 (110 – 158) U/L	35/289	1.18 (0.73 – 1.88)		0.80 (0.39 – 1.62)	
**All-cause mortality**					
Pre-KTx ALP, per doubling	**418/1180**	1.44 (1.12 – 1.84)	<0.01	1.28 (0.87 – 1.88)	0.22
Pre-KTx ALP (U/L), quartiles					
Q1, ALP 52 (46 – 57) U/L	89/302	Reference		Reference	
Q2, ALP 69 (65 – 72) U/L	107/291	1.08 (0.81 – 1.43)	0.02	0.99 (0.69 – 1.42)	0.09
Q3, ALP 87 (82 – 93) U/L	106/298	1.14 (0.86 – 1.52)	(p-trend)	0.99 (0.67 – 1.46)	(p-trend)
Q4, ALP 126 (110 – 158) U/L	116/289	1.49 (1.13 – 1.97)		1.27 (1.00 – 1.87)	

Model 1 – adjusted for age, sex; Model 2 – Model 1 + Primary kidney disease, cytomegalovirus (CMV) infection, acute allograft rejection, dialysis vintage, preemptive transplant, number of HLA mismatches, donor age and sex, living donor status, cold and warm ischemia time, history of diabetes, body mass index, serum calcium, phosphate, PTH and albumin at time of transplantation, cinacalcet and vitamin D use, , history of parathyroidectomy and decade of transplantation. Abbreviations: DGF, delayed graft function; DCGF, death censored graft failure; ALP, alkaline phosphatase; KTx, kidney transplantation; HR, hazard ratio; CI, confidence interval.

Supplementary Table 5. Association of pre-transplant (corrected) calcium levels with risk of DGF, DCGF and all-cause mortality

**Pre-transplant Serum Calcium levels (mg/dl)**	**Events**	**Model 1**		**Model 2**	
		**HR (95% CI)**	***p***	**HR (95% CI)**	***p***
**DGF**					
Pre-KTx calcium, continuous	**278/1576**	0.83 (0.43 – 1.62)	0.59	0.93 (0.43 – 2.01)	0.85
Pre-KTx calcium (mg/dl), quartiles					
Q1, Calcium 8.6 (6.6 – 9.1) mg/dl	61/393	0.91 (0.63 – 1.30)		0.90 (0.61 – 1.34)	
Q2, Calcium 9.1 (8.8 – 9.5) mg/dl	59/389	Reference	0.67	Reference	0.76
Q3, Calcium 9.5 (9.2 – 10.0) mg/dl	84/399	0.96 (0.68 – 1.34)	(p-trend)	0.98 (0.68 – 1.40)	(p-trend)
Q4, Calcium 10.1 (9.7 – 12.7) mg/dl	74/395	0.81 (0.57 – 1.15)		0.83 (0.56 – 1.24)	
**DCGF**					
Pre-KTx calcium, continuous	**150/1505**	0.57 (0.22 – 1.43)	0.23	0.32 (0.18 – 1.20)	0.09
Pre-KTx calcium (mg/dl), quartiles					
Q1, Calcium 8.6 (6.6 – 9.1) mg/dl	86/380	1.60 (0.99 – 2.58)		1.40 (0.60 – 3.29)	
Q2, Calcium 9.1 (8.8 – 9.5) mg/dl	89/374	Reference	0.24	Reference	0.85
Q3, Calcium 9.5 (9.2 – 10.0) mg/dl	107/380	1.32 (0.82 – 2.11)	(p-trend)	1.20 (0.60 – 2.41)	(p-trend)
Q4, Calcium 10.1 (9.7 – 12.7) mg/dl	148/371	1.13 (0.70 – 1.82)		1.05 (0.37 – 2.99)	
**All-cause mortality**					
Pre-KTx calcium, continuous	**433/1505**	1.12 (0.67 – 1.90)	0.66	0.88 (0.43 – 1.77)	0.72
Pre-KTx calcium (mg/dl), quartiles					
Q1, Calcium 8.6 (6.6 – 9.1) mg/dl	39/380	1.03 (0.73 – 1.39)		0.90 (0.60 – 1.33)	
Q2, Calcium 9.1 (8.8 – 9.5) mg/dl	30/374	Reference	0.83	Reference	0.80
Q3, Calcium 9.5 (9.2 – 10.0) mg/dl	41/380	1.13 (0.85 – 1.50)	(p-trend)	0.99 (0.68 – 1.46)	(p-trend)
Q4, Calcium 10.1 (9.7 – 12.7) mg/dl	40/371	1.08 (0.82 – 1.40)		0.85 (0.59 – 1.14)	

Model 1 – adjusted for age, sex; Model 2 – Model 1 + Primary kidney disease, cytomegalovirus (CMV) infection, acute allograft rejection, dialysis vintage, preemptive transplant, number of HLA mismatches, donor age and sex, living donor status, cold and warm ischemia time, history of diabetes, body mass index, plasma PTH, ALP, phosphate and albumin at time of transplantation, cinacalcet and vitamin D use, , history of parathyroidectomy and decade of transplantation. Abbreviations: DGF, delayed graft function; DCGF, death censored graft failure; KTx, kidney transplantation; HR, hazard ratio; CI, confidence interval.

Supplementary Table 6. Association of pre-transplant phosphate levels with risk of DGF, DCGF and all-cause mortality

**Pretransplant plasma phosphate levels (mg/dl)**	**Events**	**Model 1**		**Model 2**	
		**HR (95% CI)**	***p***	**HR (95% CI)**	***p***
**DGF**					
Pre-KTx phosphate, continuous	**276/1570**	1.26 (0.96 – 1.67)	0.09	1.33 (1.14 – 1.55)	<0.001
Pre-KTx phosphate (mg/dl), quartiles					
Q1, Phosphate 3.3 (1.2 – 3.8) mg/dl	73/392	1.14 (0.79 – 1.67)		0.77 (0.43 – 1.37)	
Q2, Phosphate 4.2 (3.8 – 4.7) mg/dl	63/390	Reference	0.19	Reference	<0.01
Q3, Phosphate 5.1 (4.7 – 5.6) mg/dl	62/393	0.97 (0.66 – 1.42)	(p-trend)	1.04 (0.57 – 1.87)	(p-trend)
Q4, Phosphate 6.5 (5.7 – 12.9) mg/dl	78/395	1.40 (0.97 – 2.04)		2.21 (1.21 – 4.03)	
**DCGF**					
Pre-KTx phosphate, continuous	**150/1504**	1.34 (0.98 – 1.84)	0.06	1.09 (0.96 – 1.24)	0.17
Pre-KTx phosphate (mg/dl), quartiles					
Q1, Phosphate 3.3 (1.2 – 3.8) mg/dl	30/354	0.92 (0.59 – 1.45)		0.91 (0.46 – 1.81)	
Q2, Phosphate 4.2 (3.8 – 4.7) mg/dl	27/345	Reference	0.12	Reference	0.70
Q3, Phosphate 5.1 (4.7 – 5.6) mg/dl	36/341	1.07 (0.70 – 1.63)	(p-trend)	1.25 (0.62 – 2.51)	(p-trend)
Q4, Phosphate 6.5 (5.7 – 12.9) mg/dl	57/314	1.41 (0.96 – 2.09)		1.25 (0.66 – 2.37)	
**All-cause mortality**					
Pre-KTx phosphate, continuous	**432/1504**	0.98 (0.79 – 1.21)	0.84	1.02 (0.92 – 1.11)	0.72
Pre-KTx phosphate (mg/dl), quartiles					
Q1, Phosphate 3.3 (1.2 – 3.8) mg/dl	112/354	0.72 (0.55 – 0.96)		0.69 (0.47 – 1.02)	
Q2, Phosphate 4.2 (3.8 – 4.7) mg/dl	91/345	Reference	0.13	Reference	0.18
Q3, Phosphate 5.1 (4.7 – 5.6) mg/dl	115/341	0.89 (0.68 – 1.15)	(p-trend)	1.00 (0.70 – 1.44)	(p-trend)
Q4, Phosphate 6.5 (5.7 – 12.9) mg/dl	111/314	0.94 (0.72 – 1.22)		0.91 (0.62 – 1.34)	

Model 1 – adjusted for age, sex; Model 2 – Model 1 + Primary kidney disease, cytomegalovirus (CMV) infection, acute allograft rejection, dialysis vintage, preemptive transplant, number of HLA mismatches, donor age and sex, living donor status, cold and warm ischemia time, history of diabetes, body mass index, serum PTH, ALP, calcium and albumin at time of transplantation, cinacalcet and vitamin D use, , history of parathyroidectomy and decade of transplantation. Abbreviations: DGF, delayed graft function; DCGF, death censored graft failure; KTx, kidney transplantation; HR, hazard ratio; CI, confidence interval.

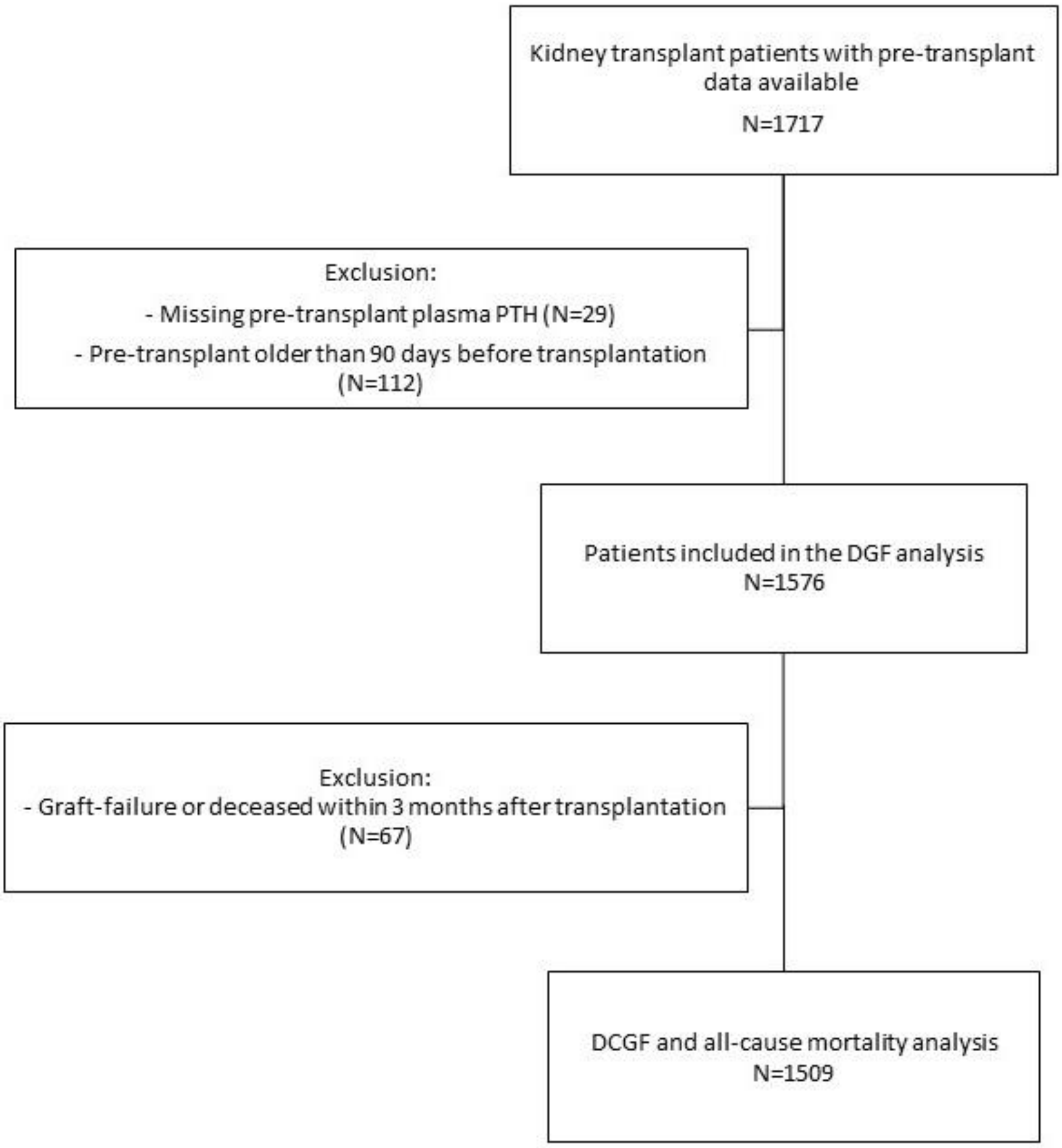

Supplementary Figure 1. Diagram flowchart of patients inclusion

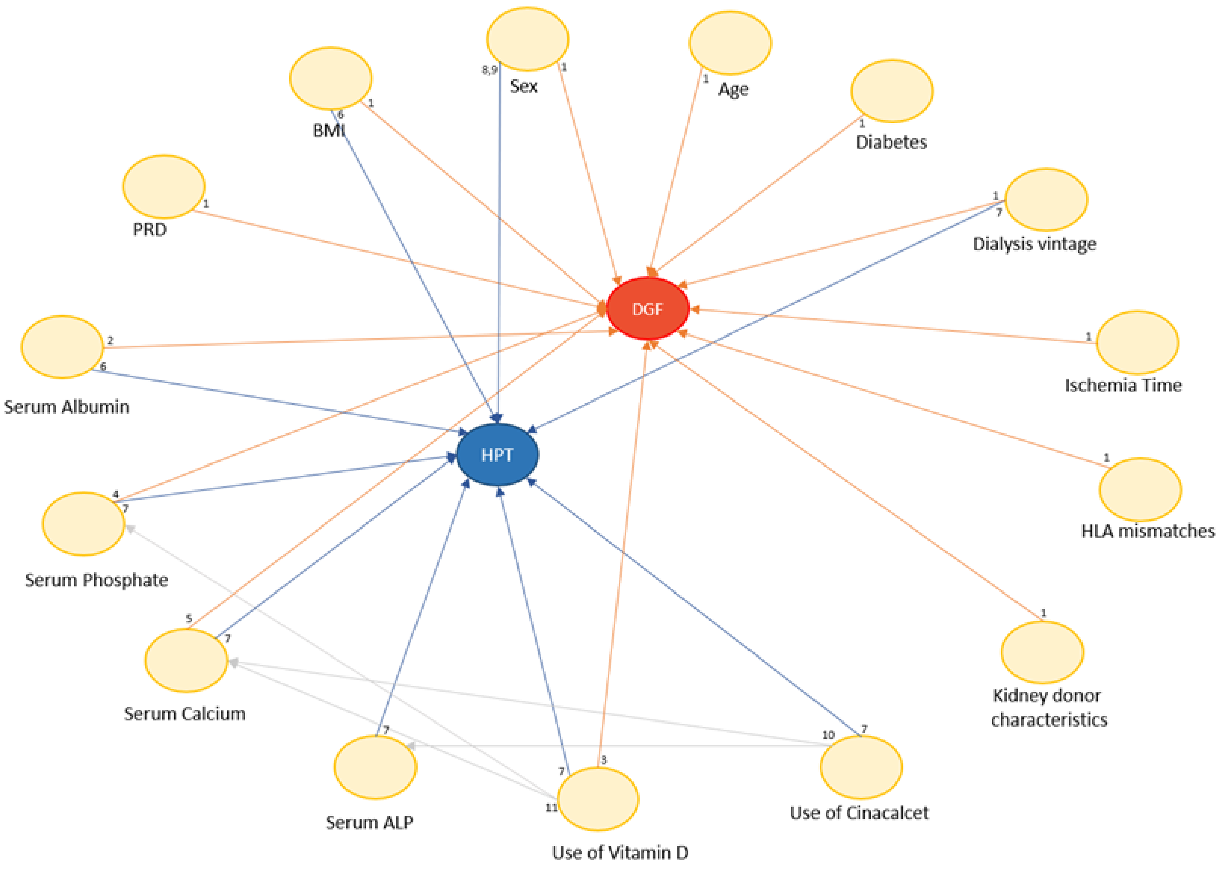

Supplementary Figure 2. A - Acyclic graph representing direct and indirect pathways between variables included in multivariable models for Delayed Graft Function (DFG). Abbreviation: HPT, hyperparathyroidism; BMI, body mass index; PRD, primary renal disease; ALP, total alkaline phosphatase; HLA, human leukocyte antigens.

References: **1.** Siedlecki A, Irish W, Brennan DC. Delayed graft function in the kidney transplant. Am J Transplant. 2011 Nov;11(11):2279 96. **2.** Yang SW, Choi JY, Kwon OJ. The impact of pretransplantation serum albumin levels on long term renal graft outcomes. Transplant Proc. 2013 May;45(4):1379 82. **3.** Koimtzis G, Stefanopoulos L, Brooker V, Geropoulos G, Chalklin CG, Gupta S, Carrington Windo E, Papaloannou M, Papavramidis TS. The Role of Vitamin D in Kidney Transplantation Outcomes: A Systematic Review. Life (Basel). 2022 Oct 20;12(10):1664. **4.** Hasanzamani B, Karimi N, Sabbagh MG, Majd HM. The Relationship Between Pre Transplant Serum Phosphorus Before Kidney Transplantation with Early Graft Dysfunction. Iran J Kidney Dis. 2021 Mar;1(2):148 154. **5.** Boom H, Mallat MJ, de Fijter JW, Paul LC, Bruijn JA, van Es LA. Calcium levels as a risk factor for delayed graft function. Transplantation. 2004 Mar 27;77(6):868 73. **6.** Disthabanchong S, Vantanasiri K, Khunapornphairote S, Chansomboon P, Buachum N, Saeseow S. Severe hyperparathyroidism is associated with nutritional impairment in maintenance hemodialysis patients. Front Nutr. 2022 Sep 13;9:933918. **7.** Cunningham J, Locatelli F, Rodriguez M. Secondary hyperparathyroidism: pathogenesis, disease progression, and therapeutic options. Clin J Am Soc Nephrol. 2011 Apr;6(4):913 21. **8.** Malberti F, Marcelli D, Conte F, Limido A, Spotti D, Locatelli F. Parathyroidectomy in patients on renal replacement therapy: an epidemiologic study. J Am Soc Nephrol. 2001 Jun;12(6):1242 1248.. **9.** Fan SL, Chan A, Raftery MJ, Yaqoob MM. Race and sex: predictors of the severity of hyperparathyroidism in peritoneal dialysis patients. Nephrology (Carlton). 2006 Feb;11(1):15 20. **10.** Moe SM, Cunningham J, Bommer J, Adler S, Rosansky SJ, Urena Torres P, Albizem MB, Guo MD, Zani VJ, Goodman WG, Sprague SM. Long term treatment of secondary hyperparathyroidism with the calcimimetic cinacalcet HCl. Nephrol Dial Transplant. 2005 Oct;20(10):2186 93. **11.** Yadav AK, Kumar V, Kumar V, Banerjee D, Gupta KL, Jha V. The Effect of Vitamin D Supplementation on Bone Metabolic Markers in Chronic Kidney Disease. J Bone Miner Res. 2018 Mar;33(3):404 409. **12.** Roodnat JI, van Gurp EA, Mulder PG, van Gelder T, de Rijke YB, de Herder WW, Kal van Gestel JA, Pols HA, Ijzermans JN, Weimar W. High pretransplant parathyroid hormone levels increase the risk for graft failure after renal transplantation. Transplantation. 2006 Aug 15;82(3):362 7. **13.** Phillips BL, Ibrahim M, Greenhall GHB, Mumford L, Dorling A, Callaghan CJ. Effect of delayed graft function on longer term outcomes after kidney transplantation from donation after circulatory death donors in the United Kingdom: A national cohort study. Am J Transplant. 2021 Oct;21(10):3346 3355. **14.** Yarlagadda SG, Coca SG, Formica RN Jr, Poggio ED, Parikh CR. Association between delayed graft function and allograft and patient survival: a systematic review and meta analysis. Nephrol Dial Transplant. 2009 Mar;24(3):1039 47. **15.** Jang Y, Park S, Lee H, Kim YH, Lee JP, Park SK, Jung IM, Ha J, Lim CS, Kim YS, Kwon H, Kim YC. Prognostic Value of Pre and Post Serum Alkaline Phosphatase Among Renal Transplant Recipients. Transplant Proc. 2022 Apr;54(3):678 684.

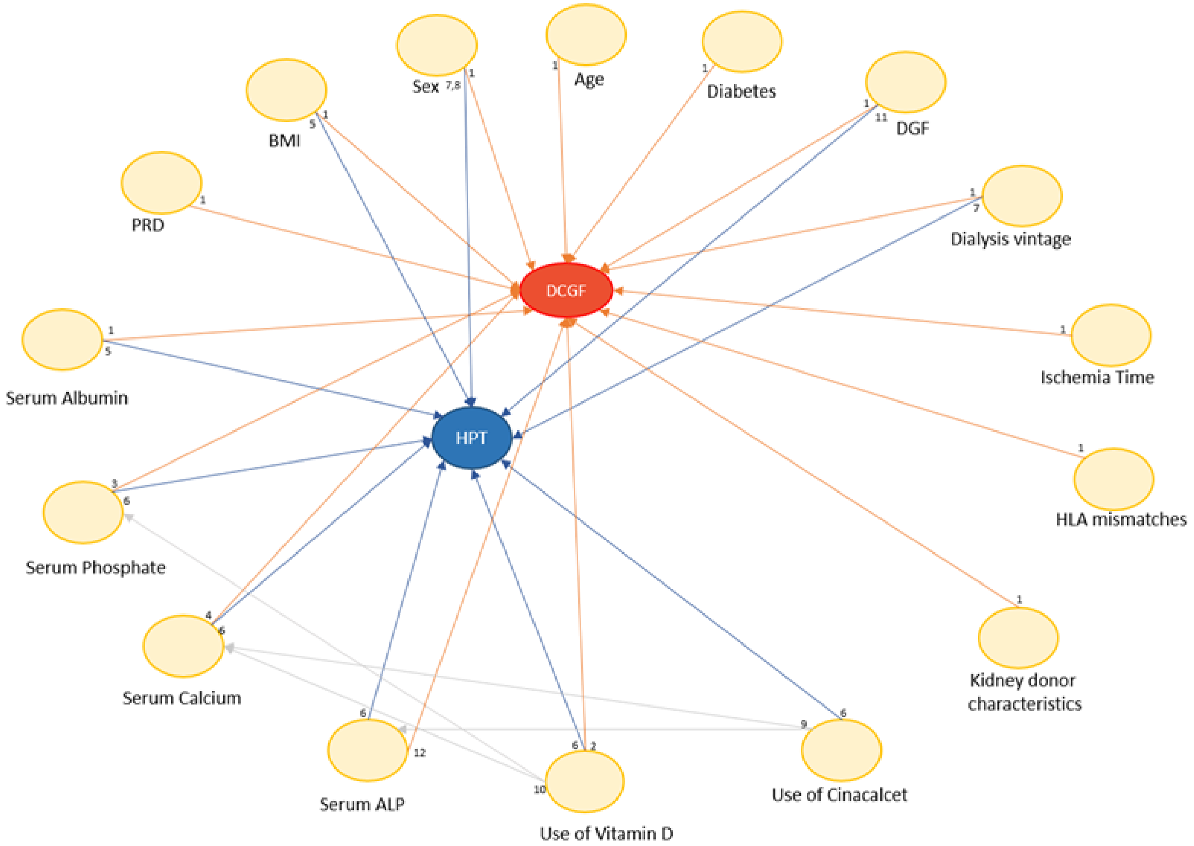

Supplementary Figure 2. B - Acyclic graph representing direct and indirect pathways between variables included in multivariable models for Death Censored Graft Failure (DCGF). Abbreviation: HPT, hyperparathyroidism; BMI, body mass index; PRD, primary renal disease; ALP, total alkaline phosphatase; HLA, human leukocyte antigens. **References:**

1. Kaboré R, Haller MC, Harambat J, Heinze G, Leffondré K. Risk prediction models for graft failure in kidney transplantation: a systematic review. Nephrol Dial Transplant. 2017 Apr 1;32(suppl_2):ii68 ii76. 2. Koimtzis G, Stefanopoulos L, Brooker V, Geropoulos G, Chalklin CG, Gupta S, Carrington Windo E, Papaloannou M, Papavramidis TS. The Role of Vitamin D in Kidney Transplantation Outcomes: A Systematic Review. Life (Basel). 2022 Oct 20;12(10):1664. 3. Sapir Pichhadze R, Parmar K, Kim SJ. Pretransplant serum phosphate levels and outcomes after kidney transplantation. J Nephrol. 2012 Nov Dec;25(6):1091 7. 4. Molnar MZ, Kovesdy CP, Mucsi I, Salusky IB, Kalantar Zadeh K. Association of pre kidney transplant markers of mineral and bone disorder with post transplant outcomes. Clinical Journal of the American Society of Nephrology. 2012;7(11):1859 1871. 5. Disthabanchong S, Vantanasiri K, Khunapornphairote S, Chansomboon P, Buachum N, Saeseow S. Severe hyperparathyroidism is associated with nutritional impairment in maintenance hemodialysis patients. Front Nutr. 2022 Sep 13;9:933918. 6. Cunningham J, Locatelli F, Rodriguez M. Secondary hyperparathyroidism: pathogenesis, disease progression, and therapeutic options. Clin J Am Soc Nephrol. 2011 Apr;6(4):913 21. 7. Malberti F, Marcelli D, Conte F, Limido A, Spotti D, Locatelli F. Parathyroidectomy in patients on renal replacement therapy: an epidemiologic study. J Am Soc Nephrol. 2001 Jun;12(6):1242 1248. 8. Fan SL, Chan A, Raftery MJ, Yaqoob MM. Race and sex: predictors of the severity of hyperparathyroidism in peritoneal dialysis patients. Nephrology (Carlton). 2006 Feb;11(1):15 20. 9. Moe SM, Cunningham J, Bommer J, Adler S, Rosansky SJ, Urena Torres P, Albizem MB, Guo MD, Zani VJ, Goodman WG, Sprague SM. Long term treatment of secondary hyperparathyroidism with the calcimimetic cinacalcet HCl. Nephrol Dial Transplant. 2005 Oct;20(10):2186 93. 10. Yadav AK, Kumar V, Kumar V, Banerjee D, Gupta KL, Jha V. The Effect of Vitamin D Supplementation on Bone Metabolic Markers in Chronic Kidney Disease. J Bone Miner Res. 2018 Mar;33(3):404 409. 11. Roodnat JI, van Gurp EA, Mulder PG, van Gelder T, de Rijke YB, de Herder WW, Kal-van Gestel JA, Pols HA, Ijzermans JN, Weimar W. High pretransplant parathyroid hormone levels increase the risk for graft failure after renal transplantation. Transplantation. 2006 Aug 15;82(3):362 7. 12. Jang Y, Park S, Lee H, Kim YH, Lee JP, Park SK, Jung IM, Ha J, Lim CS, Kim YS, Kwon H, Kim YC. Prognostic Value of Pre- and Post Serum Alkaline Phosphatase Among Renal Transplant Recipients. Transplant Proc. 2022 Apr;54(3):678 684.

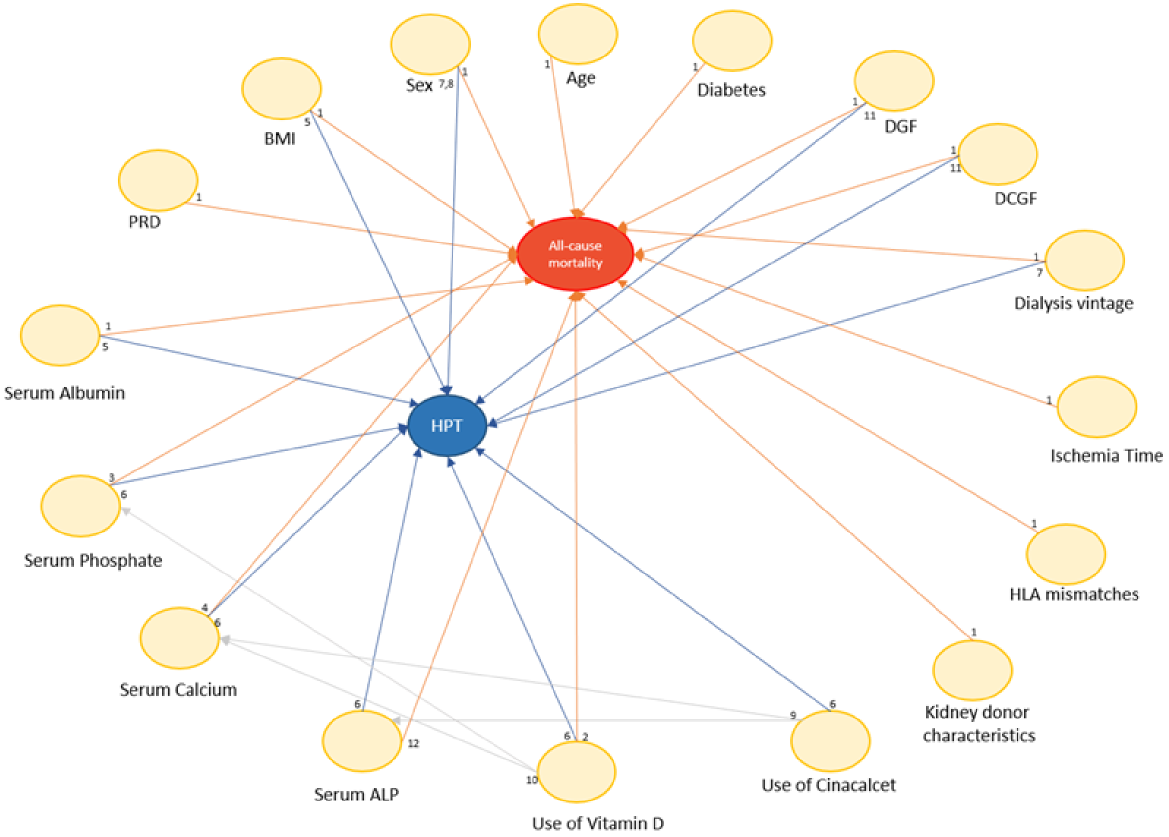

Supplementary Figure 3. C – Acyclic graph representing direct and indirect pathways between variables included in multivariable models for all cause mortality(DCGF). Abbreviation: HPT, hyperparathyroidism; BMI, body mass index; PRD, primary renal disease; ALP, total alkaline phosphatase; HLA, human leukocyte antigens; DCGF, death censored graft failure; DGF, delayed graft function. **References:** 1. Kaboré R, Haller MC, Harambat J, Heinze G, Leffondré K. Risk prediction models for graft failure in kidney transplantation: a systematic review. Nephrol Dial Transplant. 2017 Apr 1;32(suppl_2):ii68-ii76. 2. Koimtzis G, Stefanopoulos L, Brooker V, Geropoulos G, Chalklin CG, Gupta S, Carrington-Windo E, Papaloannou M, Papavramidis TS. The Role of Vitamin D in Kidney Transplantation Outcomes: A Systematic Review. Life (Basel). 2022 Oct 20;12(10):1664. 3. Sapir-Pichhadze R, Parmar K, Kim SJ. Pretransplant serum phosphate levels and outcomes after kidney transplantation. J Nephrol. 2012 Nov-Dec;25(6):1091-7. 4. Molnar MZ, Kovesdy CP, Mucsi I, Salusky IB, Kalantar-Zadeh K. Association of pre-kidney transplant markers of mineral and bone disorder with post-transplant outcomes. Clinical Journal of the American Society of Nephrology. 2012;7(11):1859-1871. 5. Disthabanchong S, Vantanasiri K, Khunapornphairote S, Chansomboon P, Buachum N, Saeseow S. Severe hyperparathyroidism is associated with nutritional impairment in maintenance hemodialysis patients. Front Nutr. 2022 Sep 13;9:933918. 6. Cunningham J, Locatelli F, Rodriguez M. Secondary hyperparathyroidism: pathogenesis, disease progression, and therapeutic options. Clin J Am Soc Nephrol. 2011 Apr;6(4):913-21. 7. Malberti F, Marcelli D, Conte F, Limido A, Spotti D, Locatelli F. Parathyroidectomy in patients on renal replacement therapy: an epidemiologic study. J Am Soc Nephrol. 2001 Jun;12(6):1242-1248. 8. Fan SL, Chan A, Raftery MJ, Yaqoob MM. Race and sex: predictors of the severity of hyperparathyroidism in peritoneal dialysis patients. Nephrology (Carlton). 2006 Feb;11(1):15-20. 9. Moe SM, Cunningham J, Bommer J, Adler S, Rosansky SJ, Urena-Torres P, Albizem MB, Guo MD, Zani VJ, Goodman WG, Sprague SM. Long-term treatment of secondary hyperparathyroidism with the calcimimetic cinacalcet HCl. Nephrol Dial Transplant. 2005 Oct;20(10):2186-93. 10. Yadav AK, Kumar V, Kumar V, Banerjee D, Gupta KL, Jha V. The Effect of Vitamin D Supplementation on Bone Metabolic Markers in Chronic Kidney Disease. J Bone Miner Res. 2018 Mar;33(3):404-409. 11. Roodnat JI, van Gurp EA, Mulder PG, van Gelder T, de Rijke YB, de Herder WW, Kal-van Gestel JA, Pols HA, Ijzermans JN, Weimar W. High pretransplant parathyroid hormone levels increase the risk for graft failure after renal transplantation. Transplantation. 2006 Aug 15;82(3):362-7. 12. Jang Y, Park S, Lee H, Kim YH, Lee JP, Park SK, Jung IM, Ha J, Lim CS, Kim YS, Kwon H, Kim YC. Prognostic Value of Pre- and Post Serum Alkaline Phosphatase Among Renal Transplant Recipients. Transplant Proc. 2022 Apr;54(3):678-684.

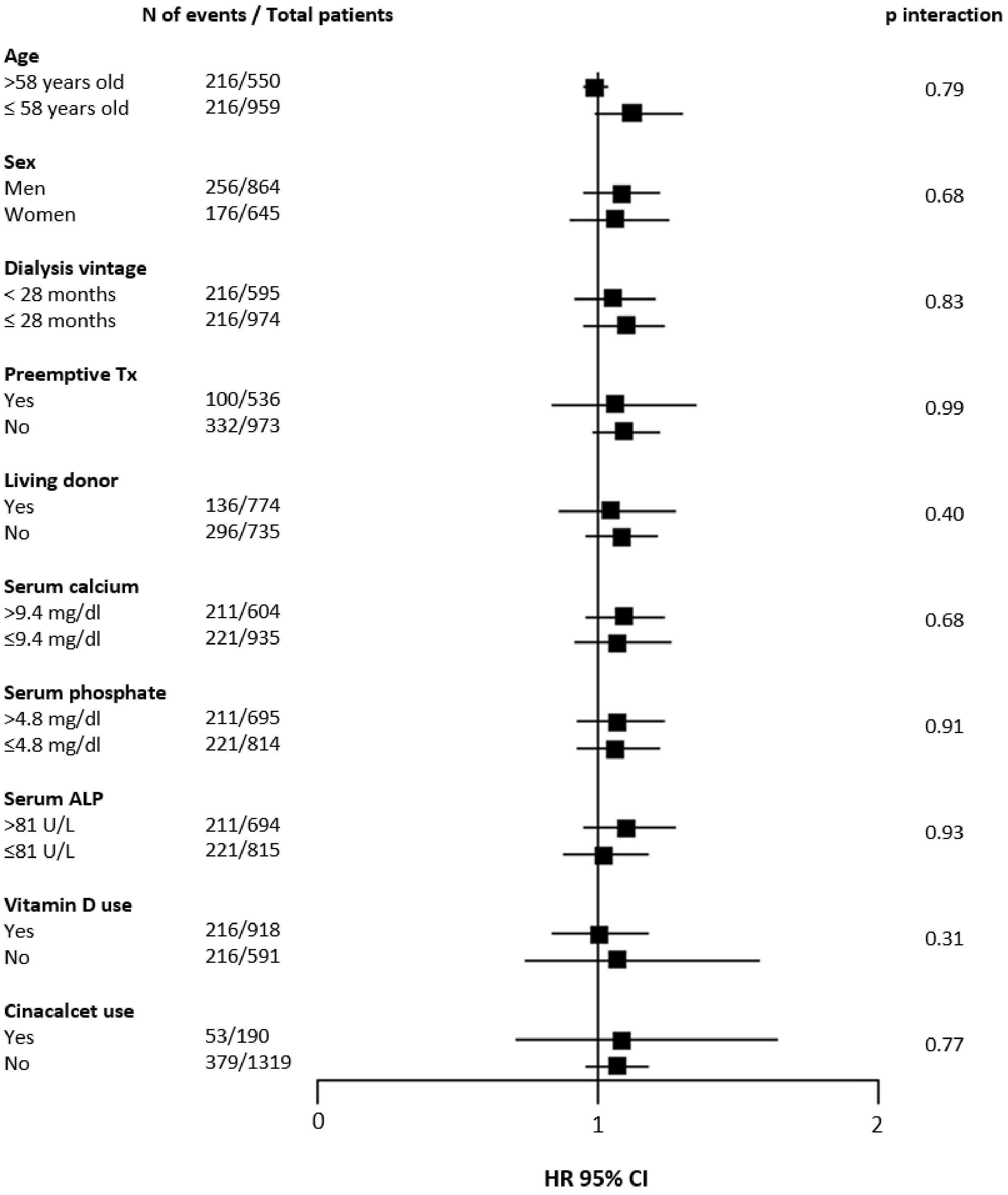

Supplementary Figure 3. Forest plot showing fully adjusted associations between pre-transplant plasma PTH levels and all-cause mortality according to subgroups and their interaction terms

STROBE Statement—checklist of items that should be included in reports of observational studies

	Item No.	Recommendation	Page No.	Relevant text from manuscript
**Title and abstract**	1	(*a*) Indicate the study's design with a commonly used term in the title or the abstract	1	
		(*b*) Provide in the abstract an informative and balanced summary of what was done and what was found	2	
**Introduction**				
Background/rationale	2	Explain the scientific background and rationale for the investigation being reported	3	
Objectives	3	State specific objectives, including any prespecified hypotheses	3	
**Methods**				
Study design	4	Present key elements of study design early in the paper	4	
Setting	5	Describe the setting, locations, and relevant dates, including periods of recruitment, exposure, follow-up, and data collection	4	
Participants	6	(*a*) *Cohort study*—Give the eligibility criteria, and the sources and methods of selection of participants. Describe methods of follow-up<br>*Case-control study*—Give the eligibility criteria, and the sources and methods of case ascertainment and control selection. Give the rationale for the choice of cases and controls<br>*Cross-sectional study*—Give the eligibility criteria, and the sources and methods of selection of participants	5	
		(*b*) *Cohort study*—For matched studies, give matching criteria and number of exposed and unexposed<br>*Case-control study*—For matched studies, give matching criteria and the number of controls per case	-	
Variables	7	Clearly define all outcomes, exposures, predictors, potential confounders, and effect modifiers. Give diagnostic criteria, if applicable	4	
Data sources/ measurement	8*	For each variable of interest, give sources of data and details of methods of assessment (measurement). Describe comparability of assessment methods if there is more than one group	4	
Bias	9	Describe any efforts to address potential sources of bias	5	
Study size	10	Explain how the study size was arrived at	4	

Continued on next page

Quantitative variables	11	Explain how quantitative variables were handled in the analyses. If applicable, describe which groupings were chosen and why	5
Statistical methods	12	(*a*) Describe all statistical methods, including those used to control for confounding	5
		(*b*) Describe any methods used to examine subgroups and interactions	6
		(*c*) Explain how missing data were addressed	6
		(*d*) *Cohort study*—If applicable, explain how loss to follow-up was addressed<br>*Case-control study*—If applicable, explain how matching of cases and controls was addressed<br>*Cross-sectional study*—If applicable, describe analytical methods taking account of sampling strategy	5
		(*e*) Describe any sensitivity analyses	9
**Results**			
Participants	13*	(a) Report numbers of individuals at each stage of study—eg numbers potentially eligible, examined for eligibility, confirmed eligible, included in the study, completing follow-up, and analysed	7
		(b) Give reasons for non-participation at each stage	-
		(c) Consider use of a flow diagram	-
Descriptive data	14*	(a) Give characteristics of study participants (eg demographic, clinical, social) and information on exposures and potential confounders	7
		(b) Indicate number of participants with missing data for each variable of interest	7
		(c) *Cohort study*—Summarise follow-up time (eg, average and total amount)	8
Outcome data	15*	*Cohort study*—Report numbers of outcome events or summary measures over time	*8*
		Case-control study—Report numbers in each exposure category, or summary measures of exposure	-
		Cross-sectional study—Report numbers of outcome events or summary measures	-
Main results	16	(*a*) Give unadjusted estimates and, if applicable, confounder-adjusted estimates and their precision (eg, 95% confidence interval). Make clear which confounders were adjusted for and why they were included	7
		(*b*) Report category boundaries when continuous variables were categorized	-
		(*c*) If relevant, consider translating estimates of relative risk into absolute risk for a meaningful time period	-

Continued on next page

Other analyses	17	Report other analyses done—eg analyses of subgroups and interactions, and sensitivity analyses	-
**Discussion**			
Key results	18	Summarise key results with reference to study objectives	10
Limitations	19	Discuss limitations of the study, taking into account sources of potential bias or imprecision. Discuss both direction and magnitude of any potential bias	11
Interpretation	20	Give a cautious overall interpretation of results considering objectives, limitations, multiplicity of analyses, results from similar studies, and other relevant evidence	11
Generalisability	21	Discuss the generalisability (external validity) of the study results	11
**Other information**			
Funding	22	Give the source of funding and the role of the funders for the present study and, if applicable, for the original study on which the present article is based	12

*Give information separately for cases and controls in case-control studies and, if applicable, for exposed and unexposed groups in cohort and cross-sectional studies.

Note: An Explanation and Elaboration article discusses each checklist item and gives methodological background and published examples of transparent reporting. The STROBE checklist is best used in conjunction with this article (freely available on the Web sites of PLoS Medicine at http://www.plosmedicine.org/, Annals of Internal Medicine at http://www.annals.org/, and Epidemiology at http://www.epidem.com/). Information on the STROBE Initiative is available at www.strobe-statement.org.
